# Supplementary material for: Protein kinase C coordinates histone H3 phosphorylation and acetylation
Source: eLife. 2015 Oct 15;4:e09886. doi: 10.7554/eLife.09886 (PMC4714974; doi:10.7554/eLife.09886)
Supplement: Supplementary fle 1. — DOI: http://dx.doi.org/10.7554/eLife.09886.018 [file elife-09886-supp1.pdf]

Supplementary Table S1; Yeast strains

Strain name	Genotype	Background	Reference/source
W303-1a	*MATa ade2-1 trp1-1 can1-100 leu2-3,112 his3-11,15 ura3-1*		
DZ2	*pGAL1-3HA-PKC1 Kan*^{R}	W303-1a	(Darieva et al., 2012)
DK186	*MATa bar1*	W303-1a	(Altman and Kellogg, 1997)
DK1690	*MATa pkc1-14::MX6*	W303-1a	(Anastasia et al, 2012)
DK1697	*MATa pkc1-21::MX6*	W303-1a	(Anastasia et al, 2012)
BY4741	*MATa his3Δ1 leu2Δ0 met15Δ0 ura3Δ0*		EUROSCARF
Y01328(*bck1Δ)*	*YJL095w::kanMX4*	BY4741	EUROSCARF
Y01490(*rtt109Δ)*	*YLL002w::kanMX4*	BY4741	EUROSCARF
DZ5	*YLL002w::kanMX4 +pAS4104* (Rtt109(WT))	BY4741	This study
DZ6	*YLL002w::kanMX4 +pAS4105* (Rtt109(T46A))	BY4741	This study
DZ7	*YLL002w::kanMX4 +pAS4106* (Rtt109(T46D))	BY4741	This study
DZ8	*YLL002w::kanMX4 +pCM188*	BY4741	This study
RMY200	*MATa ade2-101(och) his3-Δ200 lys2-801(amb) trp1-Δ901 ura3-52 hht1, hhf1::LEU2 hht2,hhf2::HIS3 plus pRM200 (CEN4 ARS1 TRP1 HHT2 HHF2)*		(Xu et al., 2005)
FXY19	RMY200 + pFX06 (HHT2-K56R)		(Xu et al., 2005)
MSY748	MATα *lys2-128δhis4-912δ leu2-3,112 ura3-52* Δ(*HHT1-HHF1*) Δ(*HHT2-HHF2*) pMS329 [URA3 HHT1 HHF1]		(Matsubara et al, 2007)
H3-T45A	MSY748 [pRS315-*HHT1(T45A)-HHF1*]		(Matsubara et al, 2007)
DZ9	H3-T45A + pAS1995 (GFP-Pkc1)		This study
